# Research Options for Controlling Zoonotic Disease in India, 2010–2015

**DOI:** 10.1371/journal.pone.0017120

**Published:** 2011-02-25

**Authors:** Nitin Sekar, Naman K. Shah, Syed Shahid Abbas, Manish Kakkar, on behalf of the Roadmap to Combat Zoonoses in India (RCZI) Initiative

**Affiliations:** 1 Department of Ecology and Evolutionary Biology, Princeton University, Princeton, New Jersey, United States of America; 2 School of Public Health, University of North Carolina, Chapel Hill, North Carolina, United States of America; 3 Public Health Foundation of India, New Delhi, India; East Carolina University School of Medicine, United States of America

## Abstract

**Background:**

Zoonotic infections pose a significant public health challenge for low- and middle-income countries and have traditionally been a neglected area of research. The Roadmap to Combat Zoonoses in India (RCZI) initiative conducted an exercise to systematically identify and prioritize research options needed to control zoonoses in India.

**Methods and Findings:**

Priority setting methods developed by the Child Health and Nutrition Research Initiative were adapted for the diversity of sectors, disciplines, diseases and populations relevant for zoonoses in India. A multidisciplinary group of experts identified priority zoonotic diseases and knowledge gaps and proposed research options to address key knowledge gaps within the next five years. Each option was scored using predefined criteria by another group of experts. The scores were weighted using relative ranks among the criteria based upon the feedback of a larger reference group. We categorized each research option by type of research, disease targeted, factorials, and level of collaboration required. We analysed the research options by tabulating them along these categories. Seventeen experts generated four universal research themes and 103 specific research options, the majority of which required a high to medium level of collaboration across sectors. Research options designated as pertaining to ‘social, political and economic’ factorials predominated and scored higher than options focussing on ecological, genetic and biological, or environmental factors. Research options related to ‘health policy and systems’ scored highest while those related to ‘research for development of new interventions’ scored the lowest.

**Conclusions:**

We methodically identified research themes and specific research options incorporating perspectives of a diverse group of stakeholders. These outputs reflect the diverse nature of challenges posed by zoonoses and should be acceptable across diseases, disciplines, and sectors. The identified research options capture the need for ‘actionable research’ for advancing the prevention and control of zoonoses in India.

## Introduction

Zoonoses have been defined as diseases and infections that are naturally transmitted between vertebrate animals and humans. Globally, zoonoses are said to account for 60% of all infectious disease pathogens and 75% of all emerging pathogens [1], [2]. The effects of zoonoses are accentuated among marginalized groups since the poor tend to have closer interactions with animals and are further removed from accessible health services. Additionally, zoonoses provide a common route for emerging infections. An analysis of recent ‘EID events’ demonstrated the increased risk of emergence of zoonotic pathogens in the Indian subcontinent. Large parts of the country were demonstrated to be global “hot spots” at high risk for emergence of pathogens from wildlife as well as domestic animals. It is suggested that human population density, human population growth, wildlife host species richness, and low latitude are predictors for the emergence of zoonotic diseases [3]. With the world's second largest human population, two biodiversity hotspots [4], and one of the world's greatest densities of tropical livestock [5], India possesses a favourable environment for the transmission of both known and novel diseases between animals and people [3], [6]. Available information in India suggests zoonotic diseases are responsible for a large burden on the public health, livestock economies, and wildlife of the country. For example, India is estimated to have the highest rabies burden in the world with more than 20,000 human deaths annually [7]; outbreaks of anthrax contracted from wild and domestic animals have led to hundreds of reported deaths [8]; the emergence of diseases from wildlife such as Nipah and Hendra viruses may be increasing [9]; and many other endemic zoonoses have been documented [10], [11], most of which disproportionately affect India's poor and marginal communities e.g. [12], [13].

Unfortunately, essential data required to mitigate the impacts of zoonotic diseases, such as nationwide estimates of burden, are not available. A targeted, multidisciplinary, and multi-sectoral effort is required to understand the risk factors for Indian zoonoses and apply the interventions necessary to control them. Such an effort is made difficult by the fragmented nature of zoonoses research and control programs in India. At the government level, the Ministry of Health and Family Welfare targets zoonotic infections in humans, the Ministry of Agriculture focuses on zoonoses in domestic animals and commodities, and the Wildlife Institute of India addresses zoonoses in wildlife; attempts to collaborate across these institutions and other educational, research, and policy organizations suffer many challenges.

The Roadmap to Combat Zoonoses in India (RCZI) initiative was launched in June 2008 with the vision of supporting and promoting integrated zoonotic disease prevention and control as a “one health”[14] concept [15]. The RCZI initiative aims to both identify key research areas for zoonoses and facilitate the linkages between the veterinary, wildlife, and public health sectors necessary to investigate them. Since there are many zoonoses research possibilities but only limited resources available to address them, it is crucial that the RCZI identify priority areas of research. Thus the RCZI decided to develop a five-year Strategic Research Agenda (SRA) to provide a blueprint for research on zoonoses over the next five years. This paper attempts to identify the research areas and more specific research options that should form the basis of the SRA through interviews of experts in the sectors related to zoonoses. The policy implications of this research is planned to be covered in more detail in a later publication.

We had the following objectives for this study: identify the primary zoonotic diseases and populations of concern; identify key gaps in our knowledge of Indian zoonoses; develop a list of research options that can fill these gaps; rank the identified options by scoring the research options using an ‘implementation perspective’; and, finally, to discern any patterns in the ranked research options that could identify the types of zoonoses-related research deemed to be more relevant to the Indian context.

## Methods

We adapted the methodology developed by the Child Health and Nutrition Research Initiative (or CHNRI, available at: http://www.chnri.org/section/publications), which has been validated in prioritization of research agendas at the national and global levels in different settings, including ones for diarrheal disease and mental health [16], [17]. The major conceptual advance in CHNRI was the recognition that health research options should be designed not only to produce new knowledge, but to provide information and tools to rapidly reduce disease burden [18]. The major methodological advance in CHNRI was two-fold. First, the systematic listing and scoring of competing research options by multiple scorers limits the influence of any one scorer's biases on the outcome. Second, by weighting scoring criteria based on the values of a reference group, the scores of the research options incorporate the priorities of the wider society in which the research should be implemented [18]. The CHNRI methods are described in detail elsewhere [18].

### Context setting and generation of research options

The initial brainstorming meeting organized by the RCZI initiative in June 2008 brought together national and international experts working on different aspects of zoonotic diseases. The meeting highlighted key knowledge gaps relating to zoonoses and provided the larger vision for the development of a Strategic Research Agenda. The Joint Working Group of RCZI met in March 2009, defining the needs and setting the broader context for the subsequent research prioritization exercise.

**Figure 1:**
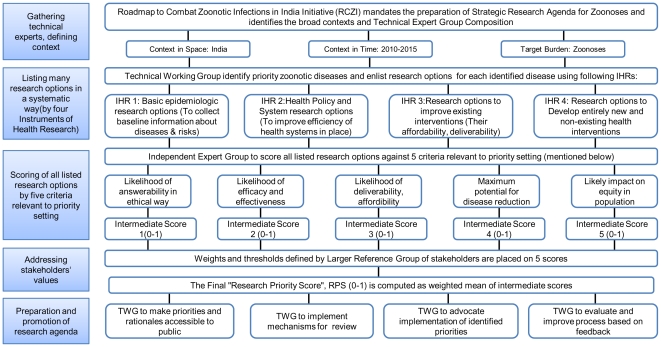
Schematic of different steps used in identification of strategic research options. Adapted from: Igor Rudan, Shams El Arifeen, Robert E. Black. A Systematic Methodology for Setting Priorities in Child Health Research Investments (In A New Approach for Systematic Priority Setting In Child Health Research Investment). Child Health and Nutrition Research Initiative (CHNRI). Bangladesh 2006.

Two groups of experts (interviewees and scorers) were selected from partner institutions of RCZI with the goal of providing inputs from different academic, policy, and sector perspectives (File S1). In order to ensure focused interviews, we asked each interviewee to identify the top five zoonotic diseases of concern in India. An exhaustive list of research options was systematically generated through interviews using an adapted version of a framework developed by CHNRI that outlines different health research areas; viz.basic epidemiological research, health policy and systems research, research to improve existing interventions, research for development of new interventions (File S2). The process was iterative, asking the interviewee to first identify key gaps in knowledge on zoonoses, and then asking for research options that could help address those gaps. Broader research options that were repeatedly identified for all diseases by many interviewees were classified as research themes (Figure 1).

### Scoring of research options

The lists of research options were randomized and individually scored by a separate group of five experts representing veterinary and public health fields and working as program managers or researchers. The experts were asked to score each of the following five criteria pre-defined by CHNRI using three yes/no questions: 1) answerability and ethics, 2) efficacy and effectiveness, 3) deliverability, affordability and sustainability, 4) maximum potential for disease burden reduction, and 5) equity in achieved disease burden reduction. Finally, the raw scores for each aforementioned criterion were weighted by the relative value accorded to each criterion by the interviewees and a larger reference group. The larger reference group was selected by emailing staff members of the Public Health Foundation of India and personal networks of authors which included scientists, students, and lay people. The final weighted score was then used to rank each research option.

We categorized the research options by the disease, affected human population groups, and animal species they targeted. We also classified options into different types of health research (basic epidemiological research, health policy and systems research, research to improve existing interventions, research for development of new interventions); based on whether they were thought to require a high, medium, or no amount of collaboration; and into different research factorials (genetic and biological, physical and environmental, ecological, social, political, economic – File S3) [19].

### Adaptation of CHNRI Methodology for Zoonoses

The original methodology developed by CHNRI dealt with relatively homogenous subject areas of diarrhea and child health conditions. We thought it necessary to make minor adaptations to make the CHNRI methodology more relevant for a heterogeneous group of little-studied conditions like zoonoses in the Indian context. Similar to another priority-setting exercise on mental health [14], an additional Instrument of Health Research focusing on basic epidemiological issues including burden estimation and risks assessment was used because of absence of good data on baseline assessments of zoonoses. Addressing the diverse challenges posed by the various zoonotic diseases and their interventions necessitated a multidisciplinary research agenda. We therefore constituted the Technical Working Group, which was composed of experts from different disciplines, sectors, and work profiles. Interviewing the members of the Technical Working Group allowed us to prepare as holistic and relevant an agenda as possible. We requested a separate group of experts to score the identified research options. While experts with in-depth, domain-specific knowledge were required for generating research options for zoonoses, the experts scoring the options had more general expertise and were thus better able to evaluate research options with the broader context in mind. Using two expert groups also enabled us to provide a level of objectivity to the prioritization exercise.

## Results

### Expert characteristics

We interviewed a diverse group of seventeen experts in order to generate an exhaustive list of research options. Of the seventeen experts, eight were primarily trained in veterinary science, three in wildlife health, four in public health, one in environmental science, and one in social science. In terms of their present position, eleven were researchers, four policy makers, and two programme managers. Fourteen of the experts work primarily at the national level (i.e. issues pertaining to India is their primary focus), and three at the international level. The experts work at national research institutes (6), multilateral agencies (4), non-profits (3), universities (2), and for the central government (2).

### Priority zoonoses

**Figure 2:**
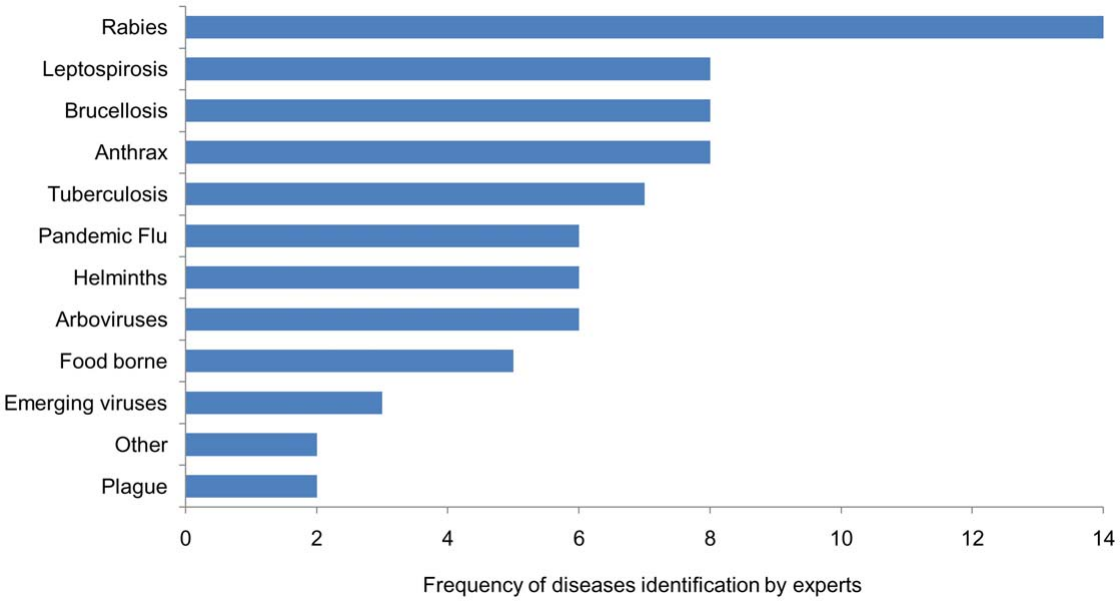
Priority zoonotic diseases in India for research as indicated from 15 experts who listed five diseases each.

Eleven major zoonotic diseases, or classes of diseases, were accorded priority status in India by our expert group. Rabies was the most frequent concern, listed by 14 of the 15 experts who provided a response. Next, anthrax, brucellosis, and leptospirosis were mentioned by eight interviewees. Tuberculosis was identified by seven experts, followed by arboviruses, helminthes, and pandemic influenzas. Remaining zoonoses included food-borne illnesses, emerging viruses, and plague (Figure 2).

### Priority areas, occupations, and populations

When the interviewees were asked about places at particular risk for zoonoses, priority areas frequently mentioned included forest fringes, northeast India, urban slums, remote villages, border areas, and disaster-prone areas. Occupations relating to farming and husbandry, the animal products industry, and animal/human/wildlife health work in forest periphery areas were identified as requiring special attention. The primary vulnerable human populations were children, tribal communities, and marginalized peoples. For behaviour change interventions, cohabitation with animals and unhygienic food practices primarily need targeting.

### Research themes

There were four broad research themes for all priority zoonoses: 1) measuring the morbidity, mortality, and economic burden of disease in humans and animals; 2) developing field diagnostics for zoonoses; 3) determining the directionality, timing, and geography of transmission between wildlife and humans and domestic animals; and 4) conducting cost-benefit, cost-effectiveness, and affordability analyses of zoonoses interventions (Table 1).

**Table 1:** Priority research themes for zoonoses in India.

1. Measure the morbidity, mortality, and economic burden of disease in humans and animals
2. Determine the spatial, temporal, and directional interactions of transmission between wildlife, humans, and domestic animals
3. Develop field diagnostics for zoonotic diseases
4. Conduct cost-benefit, cost-effectiveness, and affordability analyses of zoonoses interventions

### Weighting of scoring criteria

The interviewees along with a larger reference group ranked the five scoring criteria from 1–5 (1 being the highest) based on their assessment of their relative importance in addressing the challenge of zoonoses in India. “Deliverability, affordability and sustainability” along with “maximum potential for disease burden reduction” were seen as the most important priorities when designing zoonosis research, with average scores of 2.55. Next, “efficacy and effectiveness” scored 2.65, and “equity in achieved disease burden reduction” scored 3.61. “Answerability and ethics” had the lowest average rank at 3.65.

### Research options and their scores

In addition to four research themes, 103 research options were identified by the expert group (File S4). Some research options dealt with a particular disease, but most address challenges posed by multiple pathogens. We classified 47 research options as basic epidemiological research, 23 options as health policy and systems research, 12 options as research to improve existing interventions, and 21 options as research to develop new interventions. Of the basic epidemiological research options, 9 and 8 options respectively dealt with measuring disease burden and evaluating existing interventions. The bulk of research options (30) related to understanding risk factors. Most research options (53) dealt with social, political, and economic factors related to zoonotic diseases. 33 research options related to genetic or biological factors, 13 options to ecological concerns, and 4 options focused on physical and environmental factors. The need for collaboration between disciplines was evident with most research options thought to require a high (37) or medium (46) degree of cooperation (Table 2). Similarly, most research options applied to a range of populations (83) and commodities (62), but a number of options also target specific populations and commodities.

**Table 2:** Number of options and priority research scores by varying categories.

Row Labels	Frequency	Average Research Priority Score	Minimum Research Priority Score	Maximum Research Priority Score
**IHR**				
Health policy and systems research	23	0.81	0.64	0.94
Research to improve existing interventions	12	0.8	0.68	0.95
Basic epidemiological research	47	0.78	0.54	0.92
Research for development of new interventions	21	0.76	0.34	0.93
**Avenue**				
Evaluating existing interventions	8	0.84	0.76	0.91
Research to improve sustainability of existing interventions	6	0.83	0.73	0.92
Public health research	7	0.82	0.79	0.86
Studying system capacity to deliver efficacious interventions	11	0.82	0.71	0.91
Studying system capacity to reduce exposure to proven health risks	12	0.80	0.64	0.94
Research to improve deliverability of existing interventions	6	0.77	0.68	0.95
Understanding risk factors	30	0.77	0.54	0.92
Measuring the burden	9	0.76	0.59	0.91
Clinical research	10	0.74	0.34	0.93
Basic research	4	0.69	0.56	0.83
**Factorial**				
Social, Political, Economic	53	0.81	0.59	0.95
Ecological	13	0.79	0.55	0.91
Genetic and Biological	33	0.76	0.34	0.93
Physical and Environmental	4	0.66	0.54	0.79
**Collaboration**				
Medium	46	0.80	0.55	0.94
High	37	0.79	0.54	0.92
None	20	0.74	0.34	0.95
**Scoring criteria** *				
Answerability	103	0.80		
Efficacy	103	0.87		
Deliverability	103	0.82		
Impact	103	0.67		
Equity	103	0.76		
**Total**	**103**	**0.78**	**0.34**	**0.95**

*The scoring for the five criteria was done using an ordinal scale and all criteria had received minimum and maximum scores of 0 and 5, respectively for at least one option.

**Figure 3:**
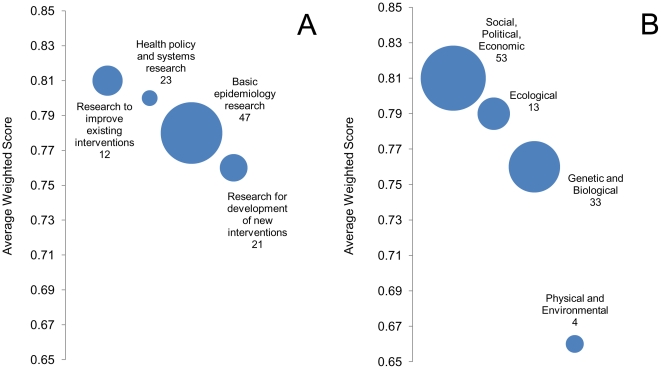
Average Score and Frequency of Research Options by (A) Instrument of Health Research (IHR); and (B) Factorial. Y Axis represents the average weighted score and the size of bubble represents frequency for each category of research option.

The research options were scored by five independent experts. The average raw score, on a scale of zero to one, was 0.78, with 0.35 as the lowest and 0.96 as the highest score. The research options scored lower on average in the categories of “maximum potential for disease reduction” and in “equity in achieved disease burden reduction” than in the other three criteria. The top fifteen research options covered a wide range of diseases, populations, commodities, types of research, and levels of collaboration (Table 3). Options related to health policy & systems research and research to improve existing interventions received higher scores on average (0.81 & 0.80, respectively) than the options focusing on epidemiologic research and research related to development of new interventions (0.78 & 0.76, respectively) (Table 2, Figure 3).

**Table 3:** Highest Scored Options for Zoonoses Research in India.

No.	Option	Answer-ability	Efficacy	Deliver-ability	Impact	Equity	Raw score	Weighted score
1	Determine the availability and prescribing policies of rabies vaccine at primary health centers and private facilities	1.00	1.00	1.00	0.80	1.00	0.96	0.95
2	Assess communication strategies for decreasing consumption of undercooked meat and promoting safe handling of carcasses to prevent anthrax	0.97	1.00	1.00	0.85	0.90	0.94	0.94
3	Develop and test vaccines for dengue	0.85	0.90	1.00	1.00	0.83	0.92	0.93
4	Define risks and mitigation options of food safety in India	0.85	0.95	0.89	1.00	0.89	0.92	0.92
5	What is the extent and mechanism of helminth drug resistance?	0.85	1.00	1.00	0.80	0.92	0.91	0.92
6	What are differences in risk factors for anthrax transmission in contrasting outbreak-prone areas?	0.92	1.00	1.00	0.80	0.83	0.91	0.91
7	Assess tuberculosis prevalence in human and animal populations in organized farms	0.85	1.00	1.00	0.80	0.91	0.91	0.91
8	Test the clinical efficacy of different antibiotics for leptospirosis treatment	0.89	1.00	0.92	0.80	0.96	0.91	0.91
9	Identify carrier bat species of nipah virus and their seasonal movement patterns	0.96	0.95	1.00	0.75	0.91	0.91	0.91
10	Do slum improvement or livelihood diversification schemes reduce the risk of exposure to zoonotic diseases?	0.88	1.00	1.00	0.69	1.00	0.91	0.91
11	Identify models and pathways for inter-sectoral collaboration and economic cooperation across sectors for zoonoses prevention and control	0.97	0.96	1.00	0.72	0.92	0.91	0.91
12	What is the impact of leptospirosis chemoprophylaxis on antibiotic resistance?	1.00	1.00	1.00	0.77	0.75	0.90	0.91
13	What are the best communication strategies to convey culling decisions to communities?	0.83	0.93	1.00	0.77	1.00	0.91	0.91
14	Conduct a risk assessment to human health from dairy-borne zoonotic diseases using Codex Alimentarius framework	0.90	1.00	0.88	0.83	0.92	0.91	0.90
15	Compare existing models for the production, purchase and distribution of rabies vaccines to identify best practices	0.96	1.00	1.00	0.65	0.90	0.90	0.90

The research priority scores for options focusing on physical and environmental themes had the average score of 0.66, whereas average scores of research options in the other three groups ranged equal to or greater than 0.76 (Table 2, Figure 3).

## Discussion

Zoonoses represent a diverse constituency. This is the first systematic attempt at identifying research priorities from an implementation perspective for zoonoses control in India. The research options were obtained from 17 interviewees representing diverse educational backgrounds, sectors and work profiles. We identified priority diseases, populations, research themes, and specific research options for zoonotic disease control in India.

### Diseases and Populations of Interest

The range of diseases identified—from viruses to helminthes, from well-known endemic diseases like rabies to the emerging Nipah virus—reflects the diversity of zoonotic challenges present in India. The populations of greatest concern were groups with frequent exposure to domestic and wild animals, such as farmers and tribal communities. As with other diseases and as in other regions [20], the identified options also reflected a special concern for the poor (urban and rural), children, and those in disaster-prone areas.

### Research Themes

Some research concerns were identified repeatedly for all diseases. These were not included as research options due to universal agreement of their necessity; ranking them would not have been informative. The emphasis on the need to generate estimates on the health, environmental, and economic impacts of zoonoses was universal. Without access to such data, allocating appropriate resources for and targeting interventions to India's vulnerable populations would be difficult. For some diseases the barrier is not technical – for example, brucellosis is well-monitored or has been eliminated in other parts of the world [21]. However, for other zoonotic diseases accurate measurement will not be possible without inexpensive diagnostics available in the field – another recurrent theme. Many zoonoses, such as leptospirosis, manifest asymptomatically or with nonspecific symptoms. Without biological confirmation, treatment and surveillance will suffer. Furthermore, many zoonoses occur predominantly in remote areas without access to laboratories or trained personnel, so tests which are simple and portable are needed. Through research on the directionality, timing, and geography of disease transmission—the third research theme—scientists will be better suited to target control interventions. Research driven by the final research theme - that of cost-benefit, cost-effectiveness and affordability of interventions, would provide policy-makers the information necessary to select and monitor zoonotic disease interventions.

### Weighting of scoring criteria

The criteria of “maximum potential to reduce the disease burden” and “deliverability, affordability, and sustainability” were ranked the highest and “answerability” was ranked the lowest by the reference group. However, the relative preference for these criteria did not substantially affect the ranking results. The top fifteen research options remained the same whether the 103 options are ranked based on raw score or by weighted score, though a few changed in their order. Assuming a representative reference group, the stakeholder community for zoonotic diseases in India preferred a research approach that balanced the five criteria rather than favouring one over the others.

Weights are most helpful when they help quantify differences in the relative importance of competing (though not necessarily conflicting) values. In this study, we found that the individuals we interviewed perceived there to be no real trade-offs amongst the criteria. Instead, the criteria were often seen to be complementary. An “answerable” research option may lead to an “efficacious and effective” intervention; “maximum disease burden reduction” may lead to “equity”, etc. This interpretation of the criteria may explain why there was no strong collective preference for any of the criteria suggested by the CHNRI methodology.

### Research options

While the high number of research options dealing with basic epidemiological research (47/103), especially understanding risk factors (30/47), underscores the need for fundamental data required to develop interventions for zoonoses, the higher scores received by the less numerous options on health systems and policy research and research to improve existing interventions reflects the immediate need for more systemic research. The findings demonstrate the relative importance of conducting applied research that helps policy makers in rational designing of policies and assists policy makers in implementing existing interventions. An alternative explanation for the abovementioned findings could be that the scoring criteria emphasise short-term results over longer term outcomes. While this provides us with a more actionable and immediate list of research concerns, it is important to also look at frequency of themes apart from research priority scores to get a complete understanding of the knowledge gaps in the area.

The evolving paradigm of interdisciplinary research removed from the traditional bio-medical centric approach is further established in our findings. Although few interviewees selected had a social science background, more than half the research options were related to the social, political, and economic domains. Significantly, the number of research options requiring high or medium interdisciplinary collaboration was larger than those occupied with a single discipline, suggesting the present “silo approach” to combating zoonoses is inadequate. Research options on social, political, and economic issues and requiring interdisciplinary collaboration also scored higher on average than their respective alternatives. This further validates the importance of a “one-health” approach that advocates active collaborations among human, veterinary, and wildlife sectors for the prevention and control of zoonotic infections [14].

### Scores

Research options received lower scores, on average, for “maximum potential for disease burden reduction” and “equity in achieved disease burden reduction” criteria than the others. That the research options received especially low marks for the former may be an artefact of the questions asked to evaluate each option on this criterion. The first question asked whether the research option being evaluated would likely lead eventually to a 5% reduction in disease burden; the second question asked the same, but for a 15% reduction; and the third asked about a 50% reduction. The scorers were reluctant to declare that any of the research options could lead to a 50% reduction or more in burden for any disease; of the 515 opportunities to answer this question, it was affirmed only 25 times (scorers could abstain). Similarly, the scorers may have been reluctant to predict equity could be achieved from any research option or its ensuing interventions. In contrast, scorers seemed more confident that a research option could be answerable or an ensuing intervention could be effective and deliverable. The deliverability scores also had the highest standard deviation, indicating this criterion may have been especially helpful in differentiating research options. The scoring methodology used by CHNRI served its stated objective of looking at research options from an implementation perspective. The Instruments of Health Research and scoring criteria favour implementable and actionable research over more esoteric themes. However in a context like that of zoonoses research in India, where limited baseline burden information is available, the methodology will need further adaptations. The next version of the zoonoses research prioritization exercise should take this into account in the study design.

The diverse fifteen highest-scoring research options reflect the many challenges posed by the zoonoses. This stands in contrast to two other studies using the CHNRI methodology, those on diarrhoea [13] and mental health [14] in developing countries, in which the highest scoring research options were dominated by health policy and systems research. In both cases, the authors suggest there may be a sufficient number of interventions available, making research on the systems necessary to distribute interventions the most critical. Since interventions and policies are available for some zoonoses in India and not for others, such a pattern is not evident in our study.

### Limitations

Zoonoses are a complex group of conditions comprising of a variety of pathogens and epidemiologic characteristics and involving complex interventions. Multisectoral involvement is necessarily required for understanding and controlling the diseases. 103 research options will not be sufficient to resolve the entire breadth of challenges posed by this complex area. This is our main limitation. However, our work highlights the information needed to deal with diseases about which so little is known.

Our use of the CHNRI methodology involved an assumption that CHNRI's five recommended scoring criteria are as applicable to the Indian zoonoses context as to the context in which they were developed, and that they represent key metrics that stakeholders would use to prioritize research options. This supposition is questionable. The challenges posed by children's health issues, for which the CHNHRI was originally developed, may be substantially different from those posed by zoonoses. It is quite possible that stakeholders engaged with Indian zoonoses would prioritize research options based on several different criteria—for instance, research that would result in technology transfer. Situationally appropriate criteria may have altered the weights determined by the reference group, allowing the final research option rankings to better reflect stakeholders' collective priorities. Developing methods for identifying situation-appropriate scoring criteria may improve the CHNRI methodology.

We were able to identify major knowledge gaps by means of the frequency with which certain subject areas were repeated by different experts. However, these subject areas did not necessarily enjoy a similar lead in scoring from a disease burden reduction point of view. This was possibly because the instruments of health research as well as scoring criteria highlighted ‘actionable’ research options in preference to basic epidemiologic research. While multiple efforts were made to decrease respondent bias by selecting a diverse group of experts and preparing detailed briefings and instructions, a possible limitation could also be the bias induced by the backgrounds and perspectives of a purposively selected group of experts. In addition, the paucity of accurate data on the prevalence and distribution of even the most common zoonoses in India means that the experts we interviewed had to rely on piecemeal data and personal experiences in identifying the regions, occupations, and populations most affected by zoonotic disease, Thus, our understanding of at-risk populations must be revised as better data becomes available.

Finally, we have not been able to incorporate the input of the communities which bear the primary burden of zoonoses into the agenda. Fortunately, we view the process of agenda setting and prioritization as dynamic. We hope to find creative ways to solicit such views as we move forward.

### Conclusions

Zoonoses are a result of complex interplay of factors that typically impact poor populations and, therefore, are a significant barrier to achieving the Millennium Development Goals in low and middle income countries. The presence of biodiversity and zoonotic ‘hot spots’ in countries such as India is accompanied with limited resources with which to tackle these conditions. In such a context, intersectoral collaborations and pooling of resources in research, policy and program implementation, as promoted by the “one health” approach will allow financially efficient and more innovative strategies for zoonoses prevention and control.

Our study proposes the development of a Strategic Research Agenda that highlights the need for multi-sectoral collaboration and for developing a systemic understanding of zoonoses prevention & control in India. In addition to providing both universal research themes and more specific research options, we have identified priority diseases and vulnerable populations that zoonoses research should target until better data on the distribution and prevalence of zoonoses allows more specific action. Research on the basic epidemiology of zoonoses; on the social, political, and economic aspects of zoonoses; and that is multidisciplinary and multisectoral should be favoured. This is the first systematic and broad-based attempt to prioritize research issues relating to zoonoses in India and will hopefully initiate discussions relating to evidence-based decision making in the country.

## Supporting Information

File S1 **Composition of Expert and Reference Groups.**
(XLS)

File S2 **Instruments of Health Research categories for understanding types of research.**
(DOC)

File S3 **Factorials examined by various research questions.**
(DOC)

File S4 **Full list of 103 research options, scores, and classifications.**
(XLS)
