# Supplementary material for: Research Options for Controlling Zoonotic Disease in India, 2010–2015
Source: PLoS One. 2011 Feb 25;6(2):e17120. doi: 10.1371/journal.pone.0017120 (PMC3045424; doi:10.1371/journal.pone.0017120)
Supplement: File S2 — Instruments of Health Research categories for understanding types of research. (DOC) [file pone.0017120.s002.doc]

**File S2- Instruments of Health Research categories for understanding types of research**

*Reference: Child Health and Nutrition Research Initiative. A new approach for systematic priority setting. 2006 Available at: http://www.chnri.org/secured/uploads/ publications/files/0535210001249198837-604_file_PRIORITY_SETTING_.pdf*

| **IHR 1: Basic epidemiological research: *defines disease burden, its components, relative risks of different underlying factors and efficacy of the available interventions to reduce the burden*** |
| --- |
| A. Measuring the burden |
| B. Understanding risk factors |
| C. Evaluating existing interventions |
|  |
| **IHR 2: Health policy and systems research: *generates new knowledge to enable more efficient use of available health care resources in reducing disease burden*** |
| D. Studying system capacity to reduce exposure to proven health risks |
| E. Studying system capacity to deliver efficacious interventions |
|  |
| **IHR 3: Research to improve existing interventions: *aims to improve deliverability, affordability and sustainability of those interventions of proven efficacy*** |
| F. Research to improve deliverability of existing interventions |
| G. Research to improve affordability of existing interventions |
| H. Research to improve sustainability of existing interventions |
|  |
| **IH4: Research for development of new interventions: *includes approaches that leads to development of new interventions, ranging from exploring the role of possible risk factors to basic molecular and genomic research that would help understand processes leading to zoonotic disease*** |
| I. Basic research |
| J. Clinical research |
| K. Public health research |
