# Supplementary material for: Research Options for Controlling Zoonotic Disease in India, 2010–2015
Source: PLoS One. 2011 Feb 25;6(2):e17120. doi: 10.1371/journal.pone.0017120 (PMC3045424; doi:10.1371/journal.pone.0017120)
Supplement: File S3 — Factorials examined by various research questions. (DOC) [file pone.0017120.s003.doc]

**Supporting Information File 3- Factorials examined by various research questions**

*Reference: Smolinski, M. S., Hamburg, M. A., Lederberg, J., ed. (2003). Microbial Threats to Health: Emergence, Detection, and Response. National Academic Press. http://www.nap.edu/catalog/10636.html*

| **Factorial** | **Examples** |
| --- | --- |
| 1. **Genetic and Biological** | microbial adaptation and change |
|  | human susceptibility to infection |
| 1. **Physical and Environmental** |  |
|  | climate and weather patterns |
|  | physical events (e.g., earthquakes, flooding) |
|  |  |
| 1. **Ecological** | changing ecosystems |
|  | development and land use |
|  |  |
| 1. **Social, Political, Economic** | human demographics and behavior |
|  | technology and industry |
|  | travel and commerce |
|  | poverty and social inequity |
|  | war and famine |
|  | bioterrorism or intent to harm |
|  | lack of political will |
|  | breakdown of public health measures |
